# Surface Acoustic Wave‐Enhanced Multi‐View Acoustofluidic Rotation Cytometry (MARC) for Pre‐Cytopathological Screening

**DOI:** 10.1002/advs.202403574

**Published:** 2024-08-13

**Authors:** Xiaoyan Zhang, Povilas Dumčius, Roman Mikhaylov, Jiangfa Qi, Mercedes Stringer, Chao Sun, Van Dien Nguyen, You Zhou, Xianfang Sun, Dongfang Liang, Dongge Liu, Bing Yan, Xi Feng, Changjun Mei, Cong Xu, Mingqian Feng, Yongqing Fu, Aled Clayton, Ruicong Zhi, Liangfei Tian, Zhiqiang Dong, Xin Yang

**Affiliations:** ^1^ Department of Electrical and Electronic Engineering, School of Engineering Cardiff University Cardiff CF24 3AA UK; ^2^ International Joint Laboratory of Biomedicine and Engineering College of Biomedicine and Health College of Life Science and Technology Huazhong Agricultural University Wuhan 430070 P. R. China; ^3^ School of Life Sciences Northwestern Polytechnical University Xi'an 710072 P. R. China; ^4^ Systems Immunity University Research Institute Cardiff University Cardiff CF14 4XN UK; ^5^ Division of Infection and Immunity Cardiff University Cardiff CF14 4XN UK; ^6^ School of Computer Science and Informatics Cardiff University Cardiff CF24 4AG UK; ^7^ Department of Engineering University of Cambridge Cambridge CB2 1PZ UK; ^8^ Department of Pathology Beijing Hospital Beijing 100730 P. R. China; ^9^ Department of Information Management Beijing Hospital Beijing 100730 P. R. China; ^10^ Department of Pathology Hubei Cancer Hospital Wuhan 430079 P. R. China; ^11^ Department of Pathology Xiangzhou District People's Hospital of Xiangyang Xiangyang 441000 P. R. China; ^12^ Faculty of Engineering and Environment Northumbria University Newcastle Upon Tyne NE1 8ST UK; ^13^ School of Medicine Cardiff University Cardiff CF14 4XN UK; ^14^ School of Computer and Communication Engineering University of Science and Technology Beijing Beijing 100083 P. R. China; ^15^ Beijing Key Laboratory of Knowledge Engineering for Materials Science Beijing 100083 P.R. China; ^16^ Department of Biomedical Engineering MOE Key Laboratory of Biomedical Engineering Zhejiang University Hangzhou 310027 P. R. China

**Keywords:** acoustofluidics, cell nuclear, cell rotation, cytopathology, surface acoustic wave

## Abstract

Cytopathology, crucial in disease diagnosis, commonly uses microscopic slides to scrutinize cellular abnormalities. However, processing high volumes of samples often results in numerous negative diagnoses, consuming significant time and resources in healthcare. To address this challenge, a surface acoustic wave‐enhanced multi‐view acoustofluidic rotation cytometry (MARC) technique is developed for pre‐cytopathological screening. MARC enhances cellular morphology analysis through comprehensive and multi‐angle observations and amplifies subtle cell differences, particularly in the nuclear‐to‐cytoplasmic ratio, across various cell types and between cancerous and normal tissue cells. By prioritizing MARC‐screened positive cases, this approach can potentially streamline traditional cytopathology, reducing the workload and resources spent on negative diagnoses. This significant advancement enhances overall diagnostic efficiency, offering a transformative vision for cytopathological screening.

## Graphical abstract

A surface acoustic wave‐enhanced multi‐view acoustofluidic rotation cytometry (MARC) device is developed for precytopathological screening. The MARC system enables multi‐angle cellular morphology analysis, enhancing the differentiation of cancerous and noncancerous cells through variations in the nuclear‐to‐cytoplasmic ratio. This innovative approach streamlines traditional cytopathology, potentially improving diagnostic efficiency and conserving medical resources.

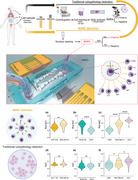

## 1 Introduction

Cytopathology, an important branch of pathology, offers diagnostic information for diseases at the cellular level, and is commonly used to diagnose diseases including cancers, inflammatory conditions, and infections.^[^ ^1^ ^]^ For example, prevention strategy of cervical cancer involves the collection of a small sample of cells from the cervix (by a smear test) for cytopathological examination to identify cells from cancer or its potential precursors.^[^ ^2^ ^]^ Cells exfoliated from a suspicious lesion through smears, scrapings, brushings, and washings, play a crucial role in disease screening. These cells are meticulously collected and transferred onto glass slides for microscopic examination, which requires highly trained pathologists. The evaluation is focused on searching cytomorphological abnormal features such as nuclear/cytoplasmic area, nuclear‐to‐cytoplasmic (N/C) ratio, and nuclear irregularity.^[^ ^3^, ^4^ ^]^ The percentage of true negative pathological results (i.e., patients do not have the disease) in smears can vary significantly depending on multiple factors such as the type of smear, the population and quality of the sample, and the expertise of the pathologist. In many cases, the majority of smears in routine screenings for human papillomavirus (HPV) yield true negative results. For instance, 60% of HPV tests for women aged 20 to 24 in the UK return negative results.^[^ ^5^ ^]^ Therefore, developing new techniques for pre‐screening biopsy specimens could significantly streamline the pathological examination process and improve the efficiency of disease diagnosis. By pre‐screening biopsy specimens, healthcare professionals can more efficiently identify high‐risk cases or areas of concern, allowing them to prioritize and focus their efforts on cases that require further examination.

Cell rotation typically refers to its rotational movement, often observed in organisms during their early development or in the context of cell migration and tissue morphogenesis.^[^ ^6^, ^7^, ^8^ ^]^ Cell rotation under external forces provides opportunities to actively manipulate cells, which facilitates 3D cell imaging and allows observation from a designated angle. The rotational motion exhibited by cells under specific conditions, such as acoustic rotation, can serve as a label‐free parameter for distinguishing different cell types and offering additional information about 3D cell morphology.^[^ ^9^ ^]^ For example, nuclear and cytoplasmic information including mitotic division have been better imaged for improving classification of suspended MCF‐7 cells through multi‐angle cell images.^[^ ^10^ ^]^ A high‐resolution 3D reconstruction of fluorescently labeled pollen grains was enabled by acoustic rotation to demonstrate and analyze their heterogeneous surface morphologies.^[^ ^11^ ^]^ Refining the techniques that manipulate the rotational dynamics of cells can offer invaluable insights, expediting the analysis and differentiation of heterogeneous cells according to their rotational characteristics.

Optical tweezers have been applied to rotate rod‐shaped bacterial cells, allowing observation of the cells from different perspectives and providing 3D subcellular structures.^[^ ^12^ ^]^ The technique showcases its versatility by trapping and spinning red blood cells, facilitating simultaneous mixing and providing various perspectives of living cells, all without subjecting them to any mechanical contact.^[^ ^13^ ^]^ Nevertheless, optical tweezers, served as a potent tool for manipulating and studying biological cells, carry the potential risk of causing cell damage under high optical intensities (>10^5^ W cm^‐2^).^[^ ^14^ ^]^ Electrical methodologies have also achieved cell rotation along three axes while simultaneously measuring their dielectric properties,^[^ ^15^ ^]^ and enables the exploration of cell clusters' proliferation, transcription, and organogenesis through induced mechanical stimulation via rotation and vibration.^[^ ^16^ ^]^ Metal‐electrode‐based dielectrophoresis has demonstrated stable self‐rotation manipulation of pigmented cells.^[^ ^17^, ^18^ ^]^ In addition, optically induced dielectrophoresis based on virtual electrode has also been reported to control the rotation of Raji, yeast, and mouse cells, with high biocompatibility and contact‐free manipulation capabilities.^[^ ^19^, ^20^, ^21^, ^22^ ^]^ Electrical manipulation is known for its low throughput and complicated experimental setup, thus limiting its applications.

Acoustofluidic technology, which utilizes surface acoustic waves (SAWs), has been increasingly applied in biomedical research, including manipulation of cells and nanoparticles,^[^ ^23^, ^24^, ^25^, ^26^ ^]^ interaction of various cell types,^[^ ^27^, ^28^ ^]^ and bioparticle separation.^[^ ^29^, ^30^, ^31^ ^]^ In contrast to optical and electric tweezers typically employed for rotating single cells, acoustofluidic devices exhibit their good capability to manipulate a large number of cells simultaneously.^[^ ^9^ ^]^ SAWs travel along the surface of a substrate, creating nanoscale mechanical vibrations on the surface while experiencing minimal acoustic energy loss within a depth of one or two wavelengths below the surface.^[^ ^32^ ^]^ Owing to their stronger interactions with surface‐bound materials, SAWs are ideal for surface‐sensitive applications such as biosensing and surface chemistry.^[^ ^33^ ^]^ Compared to bulk acoustic waves (BAWs), SAWs offer better manipulation resolution due to their higher operating frequency up to GHz ranges, and the SAW wavelengths are often comparable to or smaller than the size of typical cells, allowing for more precise control.^[^ ^34^, ^35^ ^]^ Previously microbubble‐based acoustic cell rotation technique has been developed for analyzing reproductive system pathologies and nervous system morphology in *Caenorhabditis elegans*.^[^ ^36^ ^]^ A mode‐switchable acoustofluidic device has achieved stable transportation, trapping, 3D rotation, and circular revolution of micro‐objects.^[^ ^37^ ^]^ Microbubble‐based acoustofluidics commonly involves initiating microbubbles in the device before manipulation. Alternative acoustofluidic techniques were also employed with oscillating microstructures to precisely rotate cells using acoustic microstreaming.^[^ ^38^, ^39^ ^]^ Recently, SAWs‐based techniques have been used for high‐speed direct rotation of zebrafish larvae and *C. elegans*, facilitating multispectral imaging of these model organisms and their internal structures.^[^ ^40^, ^41^ ^]^ However, although many of these acoustofluidic techniques have demonstrated in situ rotation of single objects, they did not realize multiple cell/particle manipulations with their distinctive identifications, thus offering limited throughput capabilities. Furthermore, when microbubbles are used, their quantity determines the number of cells captured and rotated. Larger cell populations necessitate longer microchannels to accommodate the physical size of each microbubble. Therefore, a device with higher throughput but a smaller footprint is desired. Herein, we present a device capable of performing multi‐view acoustofluidic rotation cytometry (MARC) for precytopathological screening. This device utilizes a meticulously designed acoustofluidic field to trap and rotate two rows of cells, simultaneously. This setup has notably increased throughput in visualizing the dynamic morphological changes of cells from multiple angles. We applied the MARC device for the first time to investigate the morphology of hepatocyte cell lines, Huh7 (cancer) and IHH (normal), from various angles. Attributed to its capability of controllable rotation of cells, MARC unveils that the cytopathological assessment, particularly the N/C ratio, is highly influenced by viewing angle. Moreover, we demonstrate that the MARC device significantly enhances sensitivity in distinguishing among different types of cells, including cancer and healthy tissue cells, compared to the traditional evaluation methods using slide‐based cytopathology. With MARC's strong ability to amplify subtle cellular differences and provide comprehensive cytomorphological information, it holds tremendous potentials for assisting pathologists in improving efficiency and conserving medical resources.

## 2 Working Mechanisms

**Figure 1:**
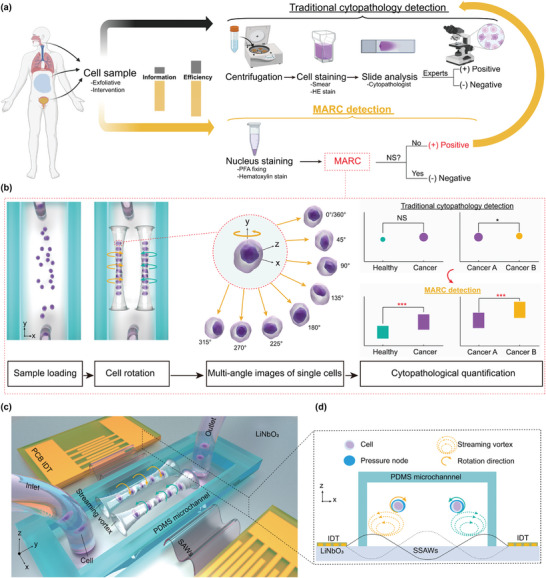
Schematic of the multi‐view acoustofluidic rotation cytometry (MARC) for pre‐cytopathological screening. a) Cell samples collected from biofluid are subjected to two analysis pathways. The traditional path involves sample centrifugation, hematoxylin and eosin (HE) staining and cytopathologist examination, yielding binary outcomes based on experience. Alternatively, the MARC detection route offers nucleus staining and muti‐view morphological analysis. Only positive MARC results (No NS: significance) prompt traditional cytopathology, enhancing screening efficiency and information depth. b) Flow chart of the working mechanism of the MARC system. c) Illustration of the MARC device, which comprises two identical printed circuit board‐based interdigital transducers (PCB‐IDTs) for producing surface acoustic waves (SAWs) and a polydimethylsiloxane (PDMS) microchannel with an inlet and an outlet for cell loading. d) SAW‐induced radiation force and microstreaming are responsible for trapping and rotating the cells, respectively. The two PCB‐IDTs generate two counter‐propagating SAWs to form a standing SAW (SSAW) yielding two pressure nodes (PNs) within the microchannel, which trap the dispersed cells to form two traces. Meanwhile, streaming vortexes produced in the microchannel drive the trapped cells to rotate opposite the streaming vortexes.

We propose a regime for applying MARC in pathological examinations (**Figure** **1**a). Cell samples obtained from human subjects are examined using the MARC device, where they are rotated to allow analysis from multiple angles. As MARC examination is a rapid process, any negative outcome does not require further evaluation using the traditional cytopathology. However, positive results, representing a proportion of all tested subjects, will undergo additional diagnosis using the traditional methods. This approach optimizes efficiency by streamlining the diagnostic process, ensuring that resources are allocated effectively to those cases warranting further scrutiny, such as those positive ones.

The MARC diagnosis process unfolds as follows (Figure 1b). By loading the cell sample into the microchannel and then activating with the SAWs, the cells will be trapped into two traces and rotated in the opposite directions. The rotational motions of the cells cause their morphological features exposed at multiple angles of observations, facilitating the characterization of multiple cellular properties corresponding to cellular status, chemical composition, or physical features. Owing to the multi‐angle images of the single cell during rotation, a more comprehensive cytopathological evaluation can be attained, potentially offering additional insights into cellular abnormalities and enhancing diagnostic assessment.

The MARC device was designed to actively induce cell rotation within the microchannel by establishing acoustic microstreaming around the cells. As shown in Figure 1c, a pair of interdigital transducers (IDTs), powered by radio frequency (RF) signals, generate identical SAWs that travel in opposite directions, effectively forming standing SAWs (SSAWs) (Figure 1d). The SSAWs exhibit a series of locations at which their amplitudes are zero or maximum, and these locations are pressure nodes (PNs) or pressure antinodes, respectively. The SAWs meet the fluid in the microchannel and leak their energy into the fluid exerting leaky acoustic waves. Generally any particles that are placed in the acoustofluidic field experience both acoustic radiation (***F***_**r**_) and streaming forces (***F***_**d**_), which are given by,^[^ ^42^, ^43^, ^44^ ^]^

(1) $$F_{r}=\;-\left(\frac{\pi P_{0}^{2}V_{c}\beta_{f}}{2\lambda }\right)\varphi \left(\beta ,\rho \right)\sin\left(2kx\right)$$

(2) $$\varphi \;\left(\beta ,\rho \right)=\frac{5\rho_{c}-2\rho_{f}}{2\rho_{c}-\rho_{f}}\;-\frac{\beta_{c}}{\beta_{f}}\;$$

(3) $$F_{d}=\;-6\pi \eta R_{p}v$$

where, *P*_0_, *V*_c_, λ, ρ_c_, ρ_f_, β_c_, β_f_, φ(β, ρ), *k*, *x*, η, *R*_c_, and ***v*** are the acoustic pressure, volume of the particle, SAW wavelength in the substrate, density of the particle, density of the fluid, compressibility of the particle, compressibility of the fluid, acoustic contrast factor, wave number, distance from a PN along the y‐axis, fluid viscosity, cell radius, and relative velocity, respectively. Details on the derivation of the radiation force and streaming velocity can be found in ref. [23] This acoustic radiation force is strongly dependent on the distance from the PN, in proportion to the volume of the particle.

The ratio of the two forces of radiation (***F***_**r**_) to the streaming forces (***F***_**d**_) can be calculated:

(4) $$\frac{F_{r}}{F_{d}}=\left(\frac{\pi P_{0}^{2}V_{c}\beta_{f}\varphi \left(\beta ,\rho \right)\sin\left(2kx\right)}{12\lambda \pi \eta R_{p}v\;}\right)\;$$

Properties of the particles such as their size, density, and compressibility, determine the above force ratio. For particles with $\frac{F_{r}}{F_{d}}>1$, the radiation force is dominant and they are attracted toward the PN. Whereas when $\frac{F_{r}}{F_{d}}<1$, the acoustic streaming is dominant and the particles are dragged to flow through micro‐circulation. It is noted that when $\frac{F_{r}}{F_{d}}=\;1$, particles are at equilibrium around the PNs, where the radiation force on a particle is balanced against the streaming force. Due to cellular heterogeneity, the cells are trapped at the equilibrium position^[^ ^36^ ^]^ where the hydrodynamic flow field produced by surrounding microstreaming induces a torque on the cells, causing them to rotate. This rotation is beneficial for thoroughly scanning the cells to capture their multi‐angle morphology while maintaining a constant microscope focus. The MARC device can simultaneously attract cells using the radiation force and rotate them with microstreaming vortices, without the need using various microstructures such as air bubbles^[^ ^36^ ^]^ or sharp edges.^[^ ^39^ ^]^

The setup of the MARC device is shown in Figure S2 (Supporting Information). The device is driven by the powered RF signals, with both incident and reflected power being monitored using two power meters. An impedance matching network is used to mitigate the impedance mismatch between the printed circuit board (PCB)‐based IDTs and an RF power amplifier.^[^ ^26^ ^]^ The technique for constructing and testing the PCB‐based IDTs has been previously introduced for various applications.^[^ ^24^, ^25^, ^26^, ^27^, ^28^ ^]^ A syringe pump is used to introduce the cell sample into the MARC device. A microscope and camera system capture sequential images, providing a rotational descriptor of the cells.

## 3 Results and Discussion

### 3.1 Optimization of Acoustofluidic Rotation

The MARC device was optimized to effectively trap the cells at the PNs for acoustic rotation driven by the induced streaming vortexes (Figure 1d). It is crucial to establish a precise and symmetric streaming vortex within the microchannel to enable simultaneous manipulation of both traces of cells. This allows for capturing the morphology of multiple cells from multiple angles simultaneously. To achieve this, the channel dimensions and acoustofluidic parameters were first studied numerically to optimize the design.

**Figure 2:**
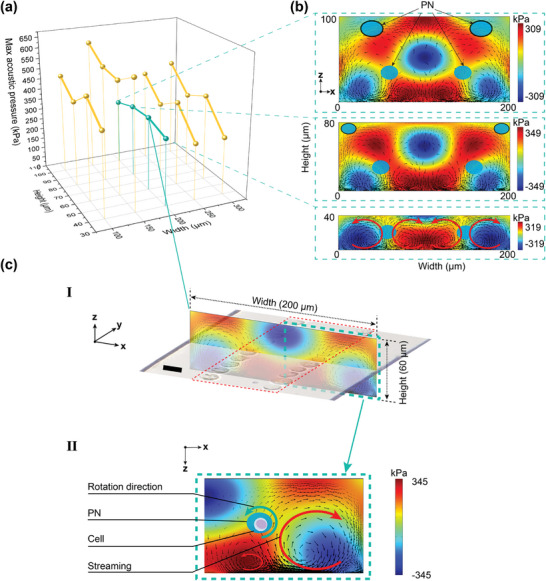
Numerical simulation of various microchannel dimensions and acoustic field in the cross‐section of the microchannel. a) The sweep of the of the maximum acoustic pressure against the dimensions of the height and width of the microchannel. The three insets in (b) show the acoustic pressure and streaming pattern for the three different channel heights with the same channel width of 200 µm. The PN is indicated by the blue oval, and the acoustic streaming direction is indicated by the black arrows. c.I) The acoustic pressure and streaming pattern for the dimension of 60 µm (height) 200 µm (width), which indicates two PN formed within the microchannel. Two cell traces formation after SAW excitation shows a good agreement with the two PNs. c.II) The zoom‐in simulation of the half microchannel dimension indicates the formation of streaming vortexes around the PN. The coexistence of the PN and streaming will lead to cell rotation in situ along the PN. The red arrows indicate the direction of regional streaming vortexes. Scale bar, 20 µm.

The width and height of the microchannel were swept from λ/2 to λ (λ ≈ 200 µm), and from λ/5 to λ/2, respectively. This range was chosen to accommodate two symmetric PNs formed along the x‐axis within the microchannel. **Figure** **2**a shows the simulation result of the maximum acoustic pressure in association with the height and width of microchannels. It is observed that a more consistent maximum acoustic pressure across different microchannel heights is achieved at a channel width of approximately 200 µm (cyan curve). This specific channel width was then chosen for further numerical analysis aimed to determine the optimal channel height.

The acoustic pressure and fluid streaming patterns for four‐channel heights, including 100, 80, 60, and 40 µm, are plotted in Figure 2b,c, showing maximum acoustic pressure of 309, 349, 345, and 319 kPa, respectively. Channel heights of 100 and 80 µm were not selected due to the presence of additional unwanted PNs near the top of the channel, as indicated by the black‐outlined blue oval regions, which are likely to trap cells against the channel wall. The channel height of 40 µm was not used either, as the fluid streaming around the PNs exhibits identical intensities but in the opposite directions, which would cancel out the cell rotation.

The channel height of 60 µm denotes a favorable balance between the PN pattern and streaming dynamics, as shown in Figure 2c. This configuration yields a symmetric distribution of PN within the MARC device, effectively trapping cells to form two distinct traces for microscopic imaging (Figure 2c.I). The simulation result is shown in Figure 2c.II, where the lateral streaming vortex displays a larger streaming velocity, leading to the in‐situ cell rotation. The fluid streaming is induced by leaky SAWs that are coupled from the vibrational substrate surface to the fluid region as presented in Figure S3 (Supporting Information). The generation of SSAWs was simulated under the condition which both IDTs were driven by the RF signals with the same phase (Δφ=0°). To further investigate the influence of phase difference, we applied various phase differences between the two IDTs, as shown in Figure S4 (Supporting Information). When applying a 90° phase difference (Δφ=90°), asymmetrical acoustic patterns form within the microchannel. When applying a 180° phase difference (Δφ=180°), although a PN is located at the center of the microchannel, the streaming flows around the PN counteract each other, negating the possibility of in situ cell rotation.

Compared with microbubble‐based acoustic rotation techniques, the MARC device overcomes the issue of temporal instability of the microbubble within the channel^[^ ^45^ ^]^ and does not require a pretreatment process before the sample loading. Additionally, cells can be accurately trapped by the two PNs located at the defined regions inside the microchannel, ensuring active and contactless manipulation of cells. This arrangement greatly enhances the convenience for multi‐angle observation.

### 3.2 High‐Throughput Cell Rotation

**Figure 3:**
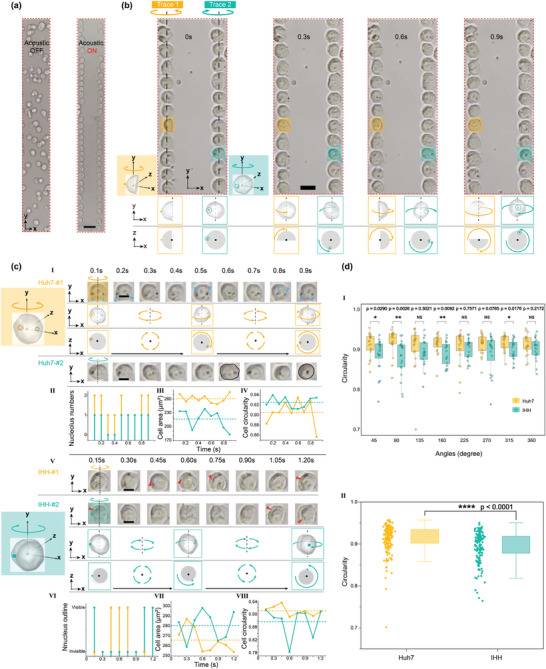
High‐throughput cell rotation manipulation. a) Cells are trapped at the two PNs to form two traces of cells when the SAW is ON, which soon to rotate in situ. The red dashed box represents the cell rotation observation field. b) All the cells in the two traces are rotating driven by the surrounding acoustic streaming. c.I–IV) Examples of Huh7 (hepatocyte cancer line) cells during rotation. The morphology parameters including the nucleolus number, cell area, and cell circularity are changing during the rotation. c.V–VIII) Example of IHH (hepatocyte cancer line) cells during rotation. The morphology parameters including the nucleus outline visibility, cell area, and cell circularity are varying during the rotation. III, IV, VII, VIII) Quantification of cell area and cell circularity during rotation process of Huh7 and IHH cell lines, respectively. The dashed lines represent the average value of the cell area and cell circularity, which is calculated based on the sum of each time‐interval quantification for the total rotation process. d) I Cell circularity comparison between Huh7 cells and IHH cells under different observation angles during rotation. II) Rotation‐based multi‐angle integrated cell circularity comparison (*n* = 20). All *p*‐values were determined using one‐way ANOVA. NS: no significance. * *p* < 0.05, ** *p* < 0.01, **** *p* < 0.001. Scale bars in (a–c) are 50 µm, 20 µm, and 10 µm, respectively.

The acoustic rotation of the cells within the MARC device was tested using two types of liver cell lines, i.e., Huh7 (cancer) and IHH (normal). To test the hypothesis that MARC can register more cellular morphology parameters, such as the number of nuclei, cell area, and cell circularity, without the need for cell staining, unstained cell samples were introduced to the MARC device resulting in an initially even dispersion in the microchannel (**Figure** **3**a, left). Upon applying RF signals to the MARC device, the acoustofluidic field is activated, exhibiting immediate trapping of cells at the two PNs (Figure 3a, right). The development of two traces of cells, where individual cells lined up end‐to‐end forming a continuous chain with no spaces between adjacent cells, significantly enhances the cell density within microfluidics. This setup positions cells at defined locations close to PNs, allowing efficient tracing rotating cells for high‐throughput multi‐view cytopathology.

The cells were rotated immediately after being trapped at the PNs, as illustrated in Figure 3b. Both traces of the cells were rotated stably near the PNs owing to the acoustic trapping effect. The microstreaming induced in opposite directions and symmetrically to the channel center leads to clockwise and counterclockwise cell rotations. Multi‐view images of each single cell, captured at different time stamps, offer highly informative morphology, revealing physical features and chemical composition at the single‐cell level. An example of rotating the Huh7 cells is demonstrated in Video S1 (Supporting Information). It is worth noting that the field of view captured in the experiment corresponds to the highlighted region (red dotted line boxes) in the simulation given in Figure 2c.I., where two traces of the cells rotate in opposite directions.

Multi‐view cellular morphology, accessible through rotation, may provide more comprehensive information to aid cytomorphology investigations. Sequential images of cells, taken at intervals ranged from 0.1 s to 0.15 s, are shown in Figure 3c, in which the dual nucleoli (indicated by blue arrows) are identified at 0.1, 0.2, 0.5, 0.8, and 0.9 s for Huh7‐#1 (Figure 3c.I) but are not visible in other views. Cell morphology varies with the observation angle, including nucleolar number, cell area, and cell circularity, all of which change during rotation (Figure 3c.II–IV). For example, the shape of cell Huh7‐#2 (outlined in black) changes from oval (0.6 s) to circular (0.9 s) at different observation angles.

For the IHH cell sample shown in Figure 3c.V and Video S3 (Supporting Information), the intermittent appearance of the nucleus during the rotation can be clearly identified without nucleus staining (indicated by red arrows). In addition, as shown in Figure 3c.VI−VIII, notable variations in the nucleus outline, cell area, and cell circularity can be clearly observed during rotation. For instance, the cell area changes by 20% from 0.3 to 1.2 s for IHH‐#1. Such changes are also found in another studies using magnetic resonance imaging (MRI) to analyze cell morphology.^[^ ^46^, ^47^ ^]^ The cell area is used as a key parameter to evaluate cell evolution, e.g., a 10.5% cell area loss was found in 50 d senescent erythrocytes.^[^ ^48^ ^]^ Additionally, cell circularity is another crucial parameter to evaluate cellular physiology state, e.g., response to drugs and other external materials. The circularity of IHH‐#2 cells changes by 18% from 0.15 to 0.6 s as shown in Figure 3c.V. A circularity reduction of 19.6%, from 0.903 to 0.726, is registered when HepG2 cells expose to 5% squaramide‐based supramolecular materials.^[^ ^49^ ^]^ The ability to detect cell area and circularity holds significant potential for advancing our understanding of cellular physiological processes.

The examples of cell morphology captured during rotation demonstrate that the MARC device successfully symmetrically trapped and rotated two traces of cells. The discovery of inconsistencies in nucleolar number, cell area, circularity, and nuclear outline within a single cell preliminarily proved the value of using MARC for multi‐angle cytology. We further applied the MARC device to compare the circularity between Huh7 and IHH cells during rotation. As shown in Figure 3d.I, no significant difference (NS) in circularity is observed at angles of 135, 225, 270, and 360 degrees. Whereas a significant difference in circularity (*p* < 0.0001) is detected between these two cell types (Figure 3d.II). This demonstrates that MARC considerably enhances the discrimination between the cancer and normal tissue cells.

### 3.3 Controllable Cell Rotation

**Figure 4:**
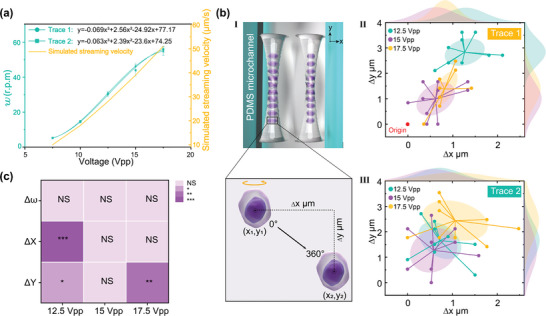
Controllable cell rotation and the stability analysis. a) The measured rotational speed and the numerical calculated streaming velocity against the amplitude of the input signal (*n* = 15). b.I) Illustration of the cell displacement on the x‐ and y‐axes. b.II, III) Distribution of the cell shift on the x‐ and y‐axis for both cell traces (*n* = 30). c) Quantification of the rotation variation between cell trace 1 and trace 2 under three input voltages. All *p*‐values were determined using one‐way ANOVA. *NS*: no significance. * *p* < 0.05, ** *p* < 0.01, *** *p* < 0.001. Scale bar, 10 µm.

The velocity of the acoustic streaming determines the rotational speed of the cells. The amplitude of the streaming velocity was controlled by the amplitude of the RF signal applied to the MARC device. The control of this speed allows capturing multi‐view morphology using the imaging system with various frame rates. **Figure** **4**a shows the rotational speeds of the cells (revolutions per minute, or r.p.m.) on both traces, as well as the calculated streaming velocity, plotted against the amplitude of the RF signal supplied to the MARC device. The rotational speeds of the cells are directly proportional to the amplitude of the RF signal. For example, increasing the amplitude from 7.5 V_PP_ to 17.5 V_PP_ results in an increase in rotational speed from ≈5.2 to ≈55 r.p.m. The representative rotation speed comparisons can be seen in Video S4 (Supporting Information) and the statistical comparison data of two cell traces are listed in Table S2 (Supporting Information). These results show that symmetric cell rotation with similar rotational speeds is created in the MARC device. As expected, the rotation angle can be predicted by using the time as their linear relationship shown in Figure S7 (Supporting Information).

The contactless feature of MARC facilitates the cell rotation taking place in a free space inside the microchannel. However, the potential cell position offset during rotational is crucial for achieving stable image capture (Figure 4b.I), which is strongly influenced by the amplitude of the RF input signal. We therefore measured the cell shift under the input signals ranging from 12.5 to 17.5 Vpp. As shown in Figure 4b.II,III, the cells on both traces drift off their starting positions for only a few microns. The average drifts under the three input signals range from ≈0.6 to ≈1.2 µm, and from 0.5 to 1 µm, respectively, on the *x*‐axis for both traces. These values indicate the minimal lateral displacements during the rotation due to the streaming effects. Similarly, the cell drift on the *y*‐axis ranges from ≈1.0 to ≈2.6 µm, and from 1.2 to 2.5 µm, respectively. While the cell displacement offset shows minimal position drift during rotation, the two cell traces exhibit inconsistent responses to input voltages along the x‐ and y‐axes. This inconsistency may stem from slight deviations in the z‐axis meaning the cell's height position is not completely stable during the rotation process. These small variations can cause tiny measurement errors when quantifying the cell's position from a top‐view (x‐y plane) while assuming a fixed position along the z‐axis.

Further analysis comparing the offset values reveals a significant variation between the two traces under 12.5 Vpp on both axes and 17.5 Vpp on the *y*‐axis (Figure 4c). The variation at 12.5 Vpp may be attributed to a weaker acoustic radiation force in trapping the cells, whereas the use of 17.5 Vpp may produce unstable acoustic streaming to drive the rotation. To ensure minimal cell displacement in situ, an input signal of 15 Vpp was used in the following manipulation, as it produced in no obvious cell drift. In comparison to optically induced and metal‐electrode‐based dielectrophoresis methods, MARC can achieve in‐situ rotation manipulation of multiple cells with minimal position drift during rotation. This feature allows imaging a group of cells without changing microscopic focus, which is crucial when using high magnification for high‐throughput cytomorphological evaluation.

### 3.4 High throughput Screening with Cell Rotation

The throughput of the current study is calculated using the formula:

(5) $$\mathrm{Throughput}\;=\frac{N}{\mathrm{RA}\times \frac{1}{\mathrm{FPS}}}\;$$

where *N* is the maximum number of cells observed in the microscope window, *FPS* is the camera frame rate, and *RA* is the number of angles captured during cell rotation.

**Figure 5:**
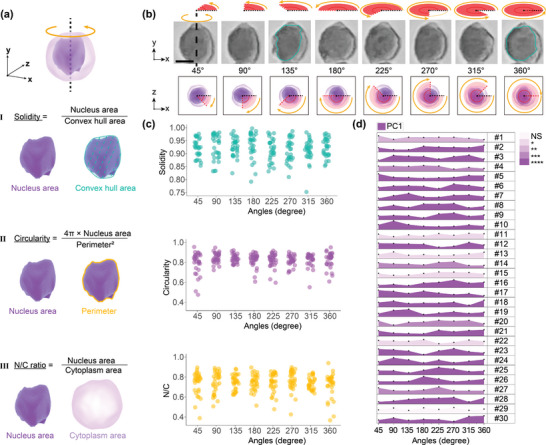
Cytopathological evaluation in the cell rotation process. a.I–III) Definition of three commonly used nuclear morphology parameters: solidity, circularity, and N/C ratio. b) Eight representative observation angle of a single hematoxylin‐stained Huh7 cell. c) Quantification of the three nuclear parameters for the Huh7 cell group (*n* = 30). d) PCA analysis of the nuclear morphology variation under different observation angles for the Huh7 cells (*n* = 30). All *p*‐values were determined using one‐way ANOVA (normally distributed) or Kruskal‐Wallis tests (non‐normally distributed). NS: no significance. * *p* < 0.05, ** *p* < 0.01, *** *p* < 0.001, **** *p* < 0.0001. Scale bar, 10 µm.

In this study:

- *N* was approximately 50, as observed with a 20× objective lens (refer to Video S2, Supporting Information).
- FPS was 30 for the camera used to validate the concept.
- RA was 8, as eight angles were captured during cell rotation (**Figure** **5**b).

The throughput was therefore estimated as:

(6) $$\frac{N}{\mathrm{RA}\times \frac{1}{\mathrm{FPS}}}=\frac{50}{8\times \frac{1}{30}}\;=\;188\;\mathrm{cells}/s$$

The throughput can be increased by using cameras with higher frame rates. For instance, a camera with 1000 fps could achieve a throughput of 6250 cells s^−1^, approaching the capacities of imaging flow cytometry.

In comparison, traditional pathology slide scanners have a throughput of about 2 min per slide, excluding slide preparation time, and can scan approximately 150 000 cells per slide.^[^ ^50^ ^]^ This corresponds to an estimated throughput of 1250 cells s^−1^. However, considering the preparation steps such as fixation, embedding, sectioning, staining, mounting, and labeling—which can take more than 24 h—the actual throughput of traditional pathology examinations might be lower than that achieved with MARC.^[^ ^10^, ^51^ ^]^ The MARC device also exhibited a much higher throughput compared to other acoustic rotation methods using microbubbles.^[^ ^11^, ^52^ ^]^ Assuming both MARC and other methods operate at the same rotation speed, the time required to capture a 360° morphology is the same. The throughput is defined by the number of cells being rotated and captured within a microscope's field of view, which is determined by the magnification used. For instance, a 20× objective lens can visualize approximately 50 cells in the field of view accommodated in the MARC device (Video S2, Supporting Information), whereas most devices applying microbubbles contain only five cells. In addition, the MARC has the capacity to attract and rotate more than two cell traces by widening the channel to accommodate more PNs, resulting in the throughput at least 10 times larger than that of microbubble methods. This higher throughput is due to the MARC device's elimination of microstructures inside the channel, allowing for a higher cell density during rotation.

### 3.5 Variation in Nuclear Solidity, Circularity, and N/C Ratio during Rotation

Cytomorphological evaluation was conducted using parameters including nucleus solidity, circularity, and N/C ratio, with their definitions illustrated in Figure 5a. It is expected that these parameters exhibit variations when measured at different angles for a given cell. To investigate the utility of the additional morphology information during rotation, the captured multi‐views of the cell were split into eight representative frames with an interval of 45°, with an example shown in Figure 5b. In this example, the nuclear morphology displays considerably different roundness;, e.g., the nucleus appears rounder at 360° compared to 135° (highlighted by the cyan outline).

We further conducted a detailed analysis of three nucleus parameters of 30 individual Huh7 cells during rotation. Each cell was quantified at eight distinct rotation angles, separated by an interval of 45°. These 30 cells were then combined to form a single group, again analyzed at the same eight rotation angles. Our analysis revealed prevalent inconsistencies in value ranges across all parameters during the group cell rotation (as shown in Figure 5c). Delving into the specifics, the solidity parameter exhibits the most substantial variation at 315°, ranging from 0.75 to 0.97. This variation suggests a broad spectrum from pronounced nucleus branching to minimal nucleus invagination. The circularity parameter, on the other hand, has its widest value which is dominant at the angle of 45°, oscillating between 0.47 and 0.95. Such a range indicates variations from highly irregular nucleus shapes to more rounded forms. Finally, the N/C ratio, a critical metric in pathology, shows marked fluctuations at angle 360°, with values ranging from 0.37 to 0.94. A high N/C ratio often indicates cellular atypia or malignancy, underscoring the importance of precise assessments.^[^ ^53^ ^]^ Inconsistencies in these measurements from different observation angles, as identified through MARC, can potentially impact diagnostic outcomes.

To better integrate the above three parameters for evaluating the nuclear morphology influenced by observation angles, we employed principal component analysis (PCA). The first principal component (PC1) accounted for a significant portion of the total variance, ranging between 40.89% and 80.07%, making it a robust indicator.^[^ ^54^ ^]^ A deeper examination of the PC1 for the 30 Huh7 cells across eight viewing angles reveals considerable variations, as depicted in Figure 5d. Remarkably, out of these samples, 29 cells exhibit significant differences (*p* < 0.05) in the PC1 across the multi‐angle views. In contrast, cell #29 maintains consistent morphology regardless of the viewing angle, as detailed in Figure S8 (Supporting Information). The observation angle plays a vital role in quantifying nuclear morphology at the single‐cell level. Details are shown in Figure S9a–c (Supporting Information), where all three parameters display significant differences under multi‐view observations, while the N/C ratio varies more than the circularity and solidity (Figure S9d, Supporting Information).

During the rotation of the 30 cells, cells #4 and #16 displayed notable fluctuations in nucleus circularity and N/C ratio values, ranging from 0.51 to 0.92 and 0.43 to 0.83, respectively. Such variations surely add complexity to cytological analysis, emphasizing the need for a thorough evaluation. For example, misshapen nuclei of fibroblast are often used as the warning signs of diseases, defined as circularity ≤ 0.65,^[^ ^55^, ^56^, ^57^ ^]^ therefore, a careful observation is required for accurate diagnosis. Similarly, distinguishing between circulating tumor cells with a N/C ratio greater than 0.8 and leukocytes with a smaller N/C ratio can be challenging, particularly in liquid biopsies where both may coexist.^[^ ^58^, ^59^ ^]^ The observation angle from the results shows that it is crucial to correctly classify these cell types.

### 3.6 Enhanced Sensitivity through Cell Rotation

The multi‐angle analysis provided by the MARC device enriches cytology by providing a more comprehensive understanding of cellular morphology, thereby aiding in overall evaluation. It is worth noting that the MARC device aims to rotate cells along the y‐axis only, unlike the rotation induced by microbubbles, which involves in rotations in both the x‐ and y‐axes. This operational difference facilitates device simplification and throughput enhancement, while still achieving a considerable sensitivity in revealing cellular morphology throughout the entire revolution. In light of these findings, we propose that MARC can enhance precytopathological screening by identifying samples marked as positive that undergo further traditional cytopathological evaluation, as illustrated in Figure 1a. This strategic approach holds the potential to greatly enhance the diagnostic efficiency.

**Figure 6:**
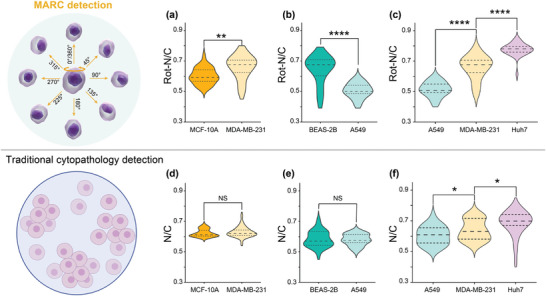
N/C‐based examination of cells. Rotation‐based N/C comparison between a) breast healthy cell MCF10A and breast cancer cell MDA‐MB‐231, between b) lung healthy cell BEAS‐2B and lung cancer cell A549, and among c) three cancer cells: lung cancer cell A549, breast cancer cell MDA‐MB‐231 and liver cancer cell Huh‐7. Traditional cytopathology detection one angle quantification based N/C comparison between d) breast healthy cell MCF10A and breast cancer cell MDA‐MB‐231, between e) lung healthy cell BEAS‐2B and lung cancer cell A549, and among (f) three cancer cells: lung cancer cell A549, breast cancer cell MDA‐MB‐231 and liver cancer cell Huh‐7. All cell lines were analyzed based on the quantification of 30 cells, per cell averaged by the N/C value of 8 angles. All *p*‐values were determined using one‐way ANOVA. NS: no significance. * *p* < 0.05, ** *p* < 0.01, **** *p* < 0.0001.

After successfully demonstrating the MARC device's capability in assessing cellular morphology from multiple angles, we further explored its potential to differentiate between tumor and normal tissue cells using this approach. Breast and lung cancer cells, along with their normal tissue counterparts, were used with the results shown in **Figure** **6**a,b.

In the case of breast cancer cells (MDA‐MB‐231), the N/C ratio measured during the rotation was found to be higher than that of epithelial cells from the mammary gland (MCF‐10A). This contrasts dramatically with the results from the conventional slide‐based examination, which shows no significant difference between cancerous and normal counterpart cells, as illustrated in Figure 6d,e. Similarly, lung cancer cells (A549) exhibited a lower N/C ratio compared to that of the lung epithelial cells (BEAS‐2B). The average N/C ratio obtained from the rotation of A549 cells was smaller than that of BEAS‐2B cells (Figure 6b), seemingly contradicting the characteristic expectation of cancer having enlarged N/C ratios due to increased chromatin content within malignant cells.^[^ ^60^ ^]^ However, this discrepancy could be attributed to alterations in their biophysical properties resulting from genetic editing during cell line development.^[^ ^61^ ^]^

These results demonstrate the MARC device's ability to offer a different perspective on N/C ratios between cancerous and noncancerous cells, providing additional data that may not be as apparent with single‐plane traditional slide‐based cytology. This Supporting Information could contribute to more informed cytological discrimination.

Another valuable application of the MARC is in phenotyping various cancer cell types, particularly in the context of differentiating cells in biofluids.^[^ ^62^, ^63^ ^]^ The N/C ratio measured during rotation (Figure 6c) reveals significant differences among A549, MDA‐MB‐231, and Huh7 cells, with the mean value of 0.51, 0.68, and 0.78, respectively. While the traditional single‐plane detection results (Figure 6f) show narrower variations in mean value at 0.61, 0.63, and 0.70, respectively.

These findings suggest that MARC clearly distinguish N/C ratios among different cell types with much better results compared to those using the standard cytological examination methods. As depicted in Figure 6, the specificity of MARC in identifying samples with significant N/C ratio variances underscores its potential as a preliminary screening tool. By directing only positively screened samples to subsequent traditional cytopathology, MARC has the great potential to optimize diagnostic efficiency.

## 4 Conclusion

In this study, we introduced the MARC device to enhance the analytical information at the single cell level. Its capability provided multi‐angle morphological insights into hepatocyte cell line Huh7 and IHH cells dramatically improved our ability to interpret cytopathological evaluations. A notable feature of this advanced cytometry is its precision, characterized by the controlled rotation speed of two cell traces, adjustable through the modulation of the device's input power. The MARC device's ability to amplify morphological variations, particularly the N/C ratio dynamics, has shown exceptional proficiency in differentiating between cancerous and noncancerous cells, surpassing traditional cytopathological methods. These findings indicate that MARC holds good promise in enhancing pre‐cytopathological screening. By focusing on samples that test positive in MARC screening for further analysis through traditional cytopathology, it could greatly improve the efficiency of the diagnostic process and optimize the use of medical resources.

## 5 Experimental Section

### Device Fabrication and Assembly

To simplify the fabrication process, the IDTs on the MARC device were manufactured by using a PCB technique.^[^ ^26^ ^]^ Briefly, a PCB patterned with a pair of gold interdigital electrodes was mechanically mounted to a piezoelectric substrate (LiNbO_3_) to form the IDT by using a jig (Figure S1, Supporting Information). The specifications of the PCB IDT and the microchannel are provided in Table S1 (Supporting Information). The Rayleigh SAW wavelength produced by the MARC device was ≈200 µm.

For the microchannel fabrication, a standard PDMS technique was employed, consistent with our previous studies.^[^ ^64^ ^]^ Initially, a 60 µm thick layer of SU‐8 photoresist was spin‐coated onto a 4 in. silicon wafer and patterned by using a mask aligner. The silicon wafer's surface was then coated with silane vapor to modify its surface properties. Subsequently, the PDMS microchannel was cast from the silicon mold with a 10:1 (w/w) mixture of PDMS base and curing agent (Sylgard 184, Dow Corning), after degassing, the PDMS was cured at 65 °C for 1 h. Finally, the cured PDMS microchannel was punched to create inlet and outlet ports for sample loading.

The assembly process for the MARC device began with the thorough cleaning of the PCB IDTs and the PDMS microchannel using isopropyl alcohol followed by deionized water. Various components of the jig as illustrated in Figure S1a (Supporting Information), including two main holders, two localized pressers, four reverse stands, and a microchannel presser, were all 3D printed. The LiNbO_3_ substrate was placed onto a customized aluminum plate to support the mechanical components. When mechanically clamping the two components, the finger electrodes of the PCB were aligned in parallel with the reference flat on the LiNbO_3_.

Four pogo pins were contacted with the bus pads on the PCB to deliver RF signals to the MARC device, eliminating the need for soldering any cables directly onto the IDTs and improved the device's pinout robustness. After the PCB IDTs were assembled, the PDMS microchannel was placed onto the LiNbO_3_. A 4 mm thick acrylic presser was then mounted onto the PDMS microchannel, providing even force distribution across the microchannel. A microchannel presser was positioned above the acrylic presser to firmly clamp the PDMS microchannel onto the LiNbO_3_. The entire MARC device was mechanically assembled, allowing for on‐demand cleaning and amendment. The combination of the PCB technique and mechanical packaging offered flexibility in constructing acoustofluidic devices.^[^ ^65^ ^]^

A polarizer (43785, Edmund Optics) was placed below the LiNbO_3_ substrate to correct the polarization, securely fitted into the hole of the aluminum plate. To enhance observation quality specifically to eliminate double‐image phenomena and achieve clearer visibility, the device was inverted to observe from the LiNbO_3_ side.

### Cell Preparation

Human hepatocytes cell lines, the Huh7 (JCRB cell bank) and IHH (JCRB cell bank),^[^ ^66^ ^]^ human lung cell lines, the human bronchial epithelial Beas‐2b (CRL‐9609, ATCC) and human non‐small cell lung cancer A549 (CCL‐185, ATCC), as well as human breast cell line such as human mammary epithelial cell line MCF‐10A (CRL‐10317, ATCC) and human breast cancer MDA‐MB‐231(HTB‐26, ATCC), were cultured in Dulbecco's modified Eagle's medium supplemented with 10% fetal bovine serum and 1% penicillin–streptomycin. The cells were passaged every 4 to 5 d upon reaching 65–80% confluency by trypsin incubation for 1–2 min, followed by phosphate‐buffered saline (PBS) washing and resuspension. The cells were then fixed with 4% paraformaldehyde for 15 min and centrifuged at 1500 rpm for 5 min to isolate the cells from the supernatant. Finally, the cells were washed again to remove the remaining paraformaldehyde.

### Nucleus Staining and Morphology Quantification

Cell nucleus staining procedure was performed by following these steps. 1) Nucleus staining: Fixed cells were suspended in 60× Mayer's hematoxylin (12603957, Fisher Scientific) in PBS to stain the nucleus for 2 min, followed by PBS washing by centrifuging at 1200 rpm for 3 min. 2) Differentiation: 2% acid alcohol was added for differentiation and immediately centrifuged the mixture at 1000 rpm for 1–2 min, and then removed the supernatant. 3) Antiblue treatment: The cell precipitate was immersed in 0.5% ammonia solution for 2 min, then centrifuged at 1000 rpm for 1–2 min, after which the supernatant was carefully removed. 4) Decolorization: 70% ethanol was added to the cell precipitate and immediately centrifuged the mixture at 1000 rpm for 1–2 min, followed by removing the supernatant. 5) Cell collection: PBS was added to resuspend the cell precipitate.

This study analyzed five nucleus shape parameters of importance for cancer diagnosis, i.e., convex hull, perimeter, area, solidity, circularity, and nuclear‐to‐cytoplasmic ratio. Solidity, defined as the ratio of the nucleus to its convex hull, was served as an indicator to assess the concavity and lobulation of the nucleus.^[^ ^67^ ^]^ Circularity, calculated as 4·p·nucleus area/nucleus perimeter,^[^ ^2^ ^]^ quantifies how closely a cell nucleus resembles as a perfect circle.^[^ ^68^ ^]^ The N/C ratio, representing the ratio of the nucleus area to the entire cytoplasmic area, provided insight into cell malignancy and atypia.^[^ ^53^ ^]^

### System Setup

Figure 1c shows the schematic of the system setup. The cells were injected into the PDMS microchannel using a syringe pump (74905‐54, Cole‐Parmer). Before loading the cells, 1% (v/v) bovine serum albumin solution was coated to the microchannel to prevent cell adhesion, at a flow rate of 5 µL min^−1^ for 30 min. All experiment data were observed and recorded through an upright microscope (OBD 127, Kern) equipped with a 20× objective lens. RF signals were generated using a signal generator (123‐6578, RS pro) and amplified by an RF power amplifier (LZY‐22+, Mini circuit). The forward and reflected powers were monitored using two power meters connected to two couplers (ZFBDC20‐62HP‐S+, Mini‐Circuits).

L‐C matching networks were designed for the PCB IDTs to minimize power reflection from the IDT. Both PCB‐IDTs, prepared using PCB technique, resulted in a Rayleigh frequency around 19.61 MHz. The reflection coefficient, *S*_11_ or *S*_22_, was used to characterize the frequency response of the IDTs. The matching network was used to optimize the signal transmission to the IDT and protected the output stage of the RF power amplifier. Figure S6 (Supporting Information) shows that the *S*_11_ for both IDTs are greatly reduced to −32.3 and −32.1 dBm, respectively, which closely resemble those of IDTs manufactured using conventional photolithography techniques.

### Numerical Analysis

To investigate the acoustofluidic field within a PDMS microchannel on the x–z plane, the finite element simulation (FEM) was conducted using COMSOL Multiphysics 6.0. The Piezoelectricity Multiphysics and Thermoviscous Acoustics Modules were employed for this study.

The model geometry consisted of ten pairs of IDT fingers (50 µm × 5 µm) with a wavelength of 200 µm, located on a 300 µm thick LiNbO_3_ wafer. The dimensions of the PDMS channel boundary were varied using the built‐in parametric sweep function, with steps of 20 µm in height (40–100 µm) and steps of 50 µm in width (100–300 µm). The liquid domain and its properties were defined as water. Initially, a frequency domain solution was performed to obtain the mechanical displacements of the piezoelectric material and the resulting acoustic pressure induced in the liquid. These acoustic pressure values were subsequently employed in the stationary solver to solve for the acoustic pressure‐induced liquid streamlines

### Analysis of Cell Rotation

Compared to many other studies involving customized algorithm for rotational analysis,^[^ ^69^, ^70^, ^71^, ^72^ ^]^ the current study used Tracker, a free video analysis and modeling tool from Open Source Physics (OSP), to perform the analysis of rotational angles and speed. In this study, videos and images were analyzed by the Tracker (OSP) and ImageJ (National Institutes of Health) software, respectively. To trace the rotation angle, 8 Huh cells were randomly selected and tracked them through two complete rotation cycles in Tracker, recording the timestamp at each 45° interval. For determining the rotation speed, 15 randomly selected Huh7 cell rotation cycles were analyzed, each representing a full 360° rotation, from the two cell traces. Using Tracker software, the number of frames (*x*_n_ − *x*_0_) was counted for a 360° rotation. The cell rotation speed ω can be calculated using the following equations,^[^ ^70^ ^]^

(7) $$\omega \;=\;\frac{60(x_{n}-x_{0})}{\mathrm{FPS}}$$

where FPS represents the frame rate of the camera capturing the rotation.

Additionally, the measurement of parameters such as nucleus area, nucleus convex hull area, nucleus perimeter, cytoplasm area, cell area, cell perimeter, and N/C ratio is typically accomplished through interactive image analysis and segmentation techniques made available in ImageJ. 1) Nucleus Area: the pixel area occupied was interactively identified and measured by the nucleus in the frame image, often after thresholding to separate the nucleus from the background. 2) Nucleus Convex Hull Area: the area of the smallest convex shape was outlined that can entirely enclose the nucleus. ImageJ calculated this by creating a convex hull around the segmented nucleus and measuring its area. 3) Nucleus Perimeter: This measured the total length of the boundary around the nucleus. ImageJ determined this by tracing the outline of the segmented nucleus. 4) Cytoplasm Area: The cytoplasm area was measured by subtracting the nucleus area from the total cell area after segmenting the cell and the nucleus separately. 5) Cell Area: This parameter is the total pixel area occupied by the entire cell, including both the nucleus and the cytoplasm. ImageJ calculates this by segmenting the cell from the background. 6) Cell Perimeter: Similar to the nucleus perimeter, this is the total length of the boundary around the entire cell outlined around the segmented cell. 7) N/C Ratio: The N/C ratio was computed by dividing the intensity measurement of the nuclear region of interest (ROI) by that of the cytoplasmic ROI (Figure 5a).

### Statistics Analysis

In this case, principal component analysis (PCA)^[^ ^73^ ^]^ was conducted after z‐score standardization of cell nucleus solidity, circularity, and N/C indices. The first coordination of PCA (PC1) accounted for the most of total variances was used as the reduced set of nucleus shape index. One‐way ANOVA (normally distributed) or Kruskal‐Wallis tests (non‐normally distributed) were performed to evaluate the statistical significance of differences. *, **, ***, and **** indicate *p* < 0.05, 0.01, 0.001, and 0.0001 between the conditions, respectively, and NS indicates statistically no significant difference between the conditions. All statistical results were plotted using Origin (OriginPro 2023, OriginLab).

## Conflict of Interest

The authors declare no conflict of interest.

## Author Contributions

X.Z.: conceptualization, investigation, data analysis, device design, performed the experiments, writing‐original draft. P.D.: investigation, numerical simulation, methodology‐device fabrication, writing‐review and editing. R.M.: investigation, methodology‐device fabrication. J.Q.: methodology‐cell culture and fixing, suspension cell staining. M.S.: writing‐review and editing. C.S.: resources. V.D.N.: methodology‐cell culture and fixing. Y.Z.: writing‐review and editing. X.S.: writing‐review and editing. D.L.: resources. D.L.: investigation, supervision. B.Y.: investigation. X.F.: methodology‐cell staining. C.M.: methodology‐cell staining. C.X.: resources. M.F.: resources. Y.F.: investigation, writing‐review and editing. R. Z.: resources. L.T.: resources. A.C.: writing‐review and editing. Z.D.: investigation, supervision, resources, writing‐review and editing. X.Y.: conceptualization, investigation, supervision, funding acquisition, resources, writing‐review and editing.

## Supporting information

Supporting Information

Supplemental Video 1

Supplemental Video 2

Supplemental Video 3

Supplemental Video 4

## Data Availability

The data that support the findings of this study are available from the corresponding author upon reasonable request.
