# Supplementary material for: Surface Acoustic Wave‐Enhanced Multi‐View Acoustofluidic Rotation Cytometry (MARC) for Pre‐Cytopathological Screening
Source: Adv Sci (Weinh). 2024 Aug 13;11(39):2403574. doi: 10.1002/advs.202403574 (PMC11497091; doi:10.1002/advs.202403574)
Supplement: Supplementary file 1 — Supporting Information [file ADVS-11-2403574-s004.docx]

Supporting Information for:

**Surface Acoustic Wave-Enhanced Multi-View Acoustofluidic Rotation Cytometry (MARC) for Pre-Cytopathological Screening**

Xiaoyan Zhang^1.2^, Povilas Dumčius^1^, Roman Mikhaylov^1^, Jiangfa Qi^2^, Mercedes Stringer^1^, Chao Sun^3^, Van Dien Nguyen^4,5^, You Zhou^4,5^, Xianfang Sun^6^, Dongfang Liang^7^, Dongge Liu^8^*, Bing Yan^9^, Xi Feng^10^, Changjun Mei^11^, Cong Xu^11^, Mingqian Feng^2^, Yongqing Fu^12^, Aled Clayton^13^, Ruicong Zhi^14,15^, Liangfei Tian^16^, Zhiqiang Dong^2^*, Xin Yang^1^*

^1^ Department of Electrical and Electronic Engineering, School of Engineering, Cardiff University, Cardiff CF24 3AA, UK

^2^ International Joint Laboratory of Biomedicine and Engineering, College of Biomedicine and Health, College of Life Science and Technology, Huazhong Agricultural University, Wuhan 430070, P.R. China

^3^ School of Life Sciences, Northwestern Polytechnical University, Xi’an 710072, P.R. China

^4^ Systems Immunity University Research Institute, Cardiff University, Cardiff CF14 4XN, UK

^5^ Division of Infection and Immunity, Cardiff University, Cardiff CF14 4XN, UK

^6^ School of Computer Science and Informatics, Cardiff University, Cardiff CF24 4AG, UK

^7^ Department of Engineering, University of Cambridge, Cambridge CB2 1PZ, UK

^8^ Department of Pathology, Beijing Hospital, Beijing 100730, P.R. China

^9^ Department of Information Management, Beijing Hospital, Beijing 100730, P.R. China

^10^ Department of Pathology, Hubei Cancer Hospital, Wuhan 430079, P.R. China

^11^ Department of Pathology, Xiangzhou District People's Hospital of Xiangyang, Xiangyang 441000, P.R. China

^12^ Faculty of Engineering and Environment, Northumbria University, Newcastle Upon Tyne NE1 8ST, UK

^13^ School of Medicine, Cardiff University, Cardiff CF14 4XN, UK

^14^ School of Computer and Communication Engineering, University of Science and Technology Beijing, Beijing 100083, P.R. China

^15^ Beijing Key Laboratory of Knowledge Engineering for Materials Science, Beijing 100083, P.R. China

^16^ Department of Biomedical Engineering, MOE Key Laboratory of Biomedical Engineering, Zhejiang University, Hangzhou 310027, P.R. China

^*^Corresponding author, email: [liudongge2231@bjhmoh.cn](mailto:liudongge2231@bjhmoh.cn), [dongz@mail.hzau.edu.cn](mailto:dongz@mail.hzau.edu.cn), [yangx26@cardiff.ac.uk](mailto:yangx26@cardiff.ac.uk)

**
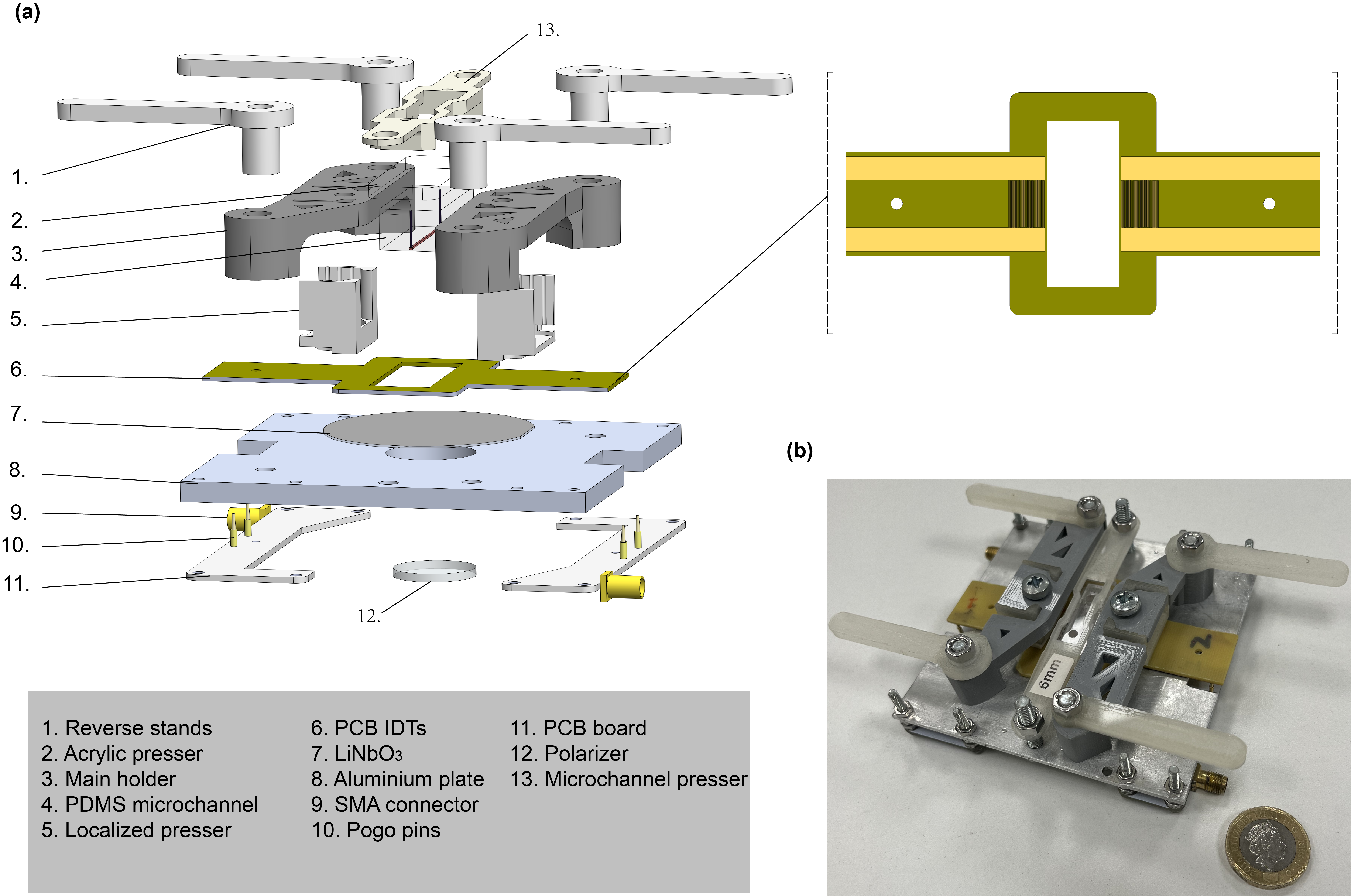
**

**Figure S1. The components and assembly of the MARC device. (a)** 3D exploded view of the MARC device. **(b)** Real image of the assembled MARC device.





**Figure S2. Schematic of the MARC experiment setup.**


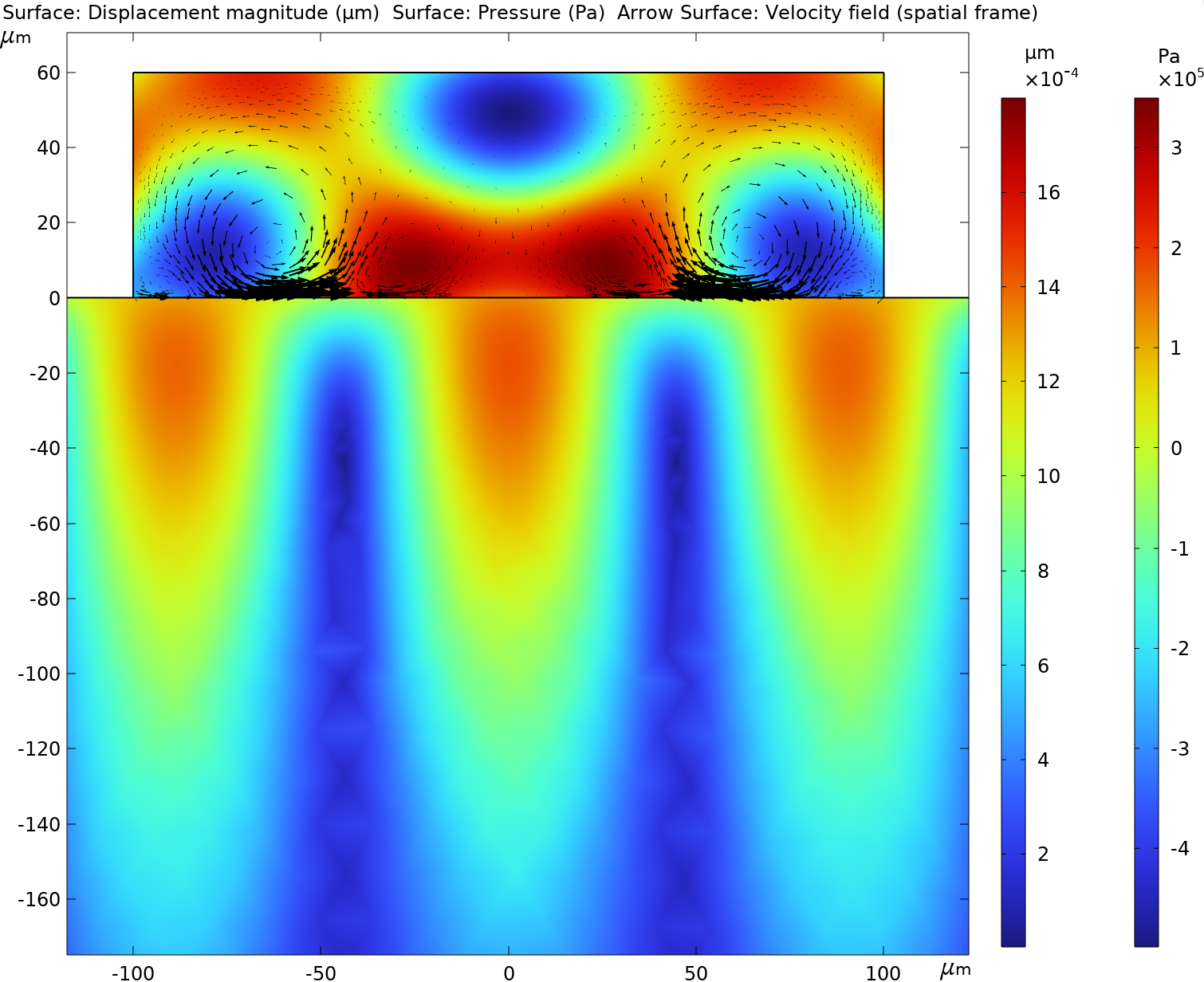


**Figure S3. Numerical simulation coupling of acoustics from the vibrational surface to the fluid region for the microchannel dimensional of 60 μm (height) 200 μm (width).**


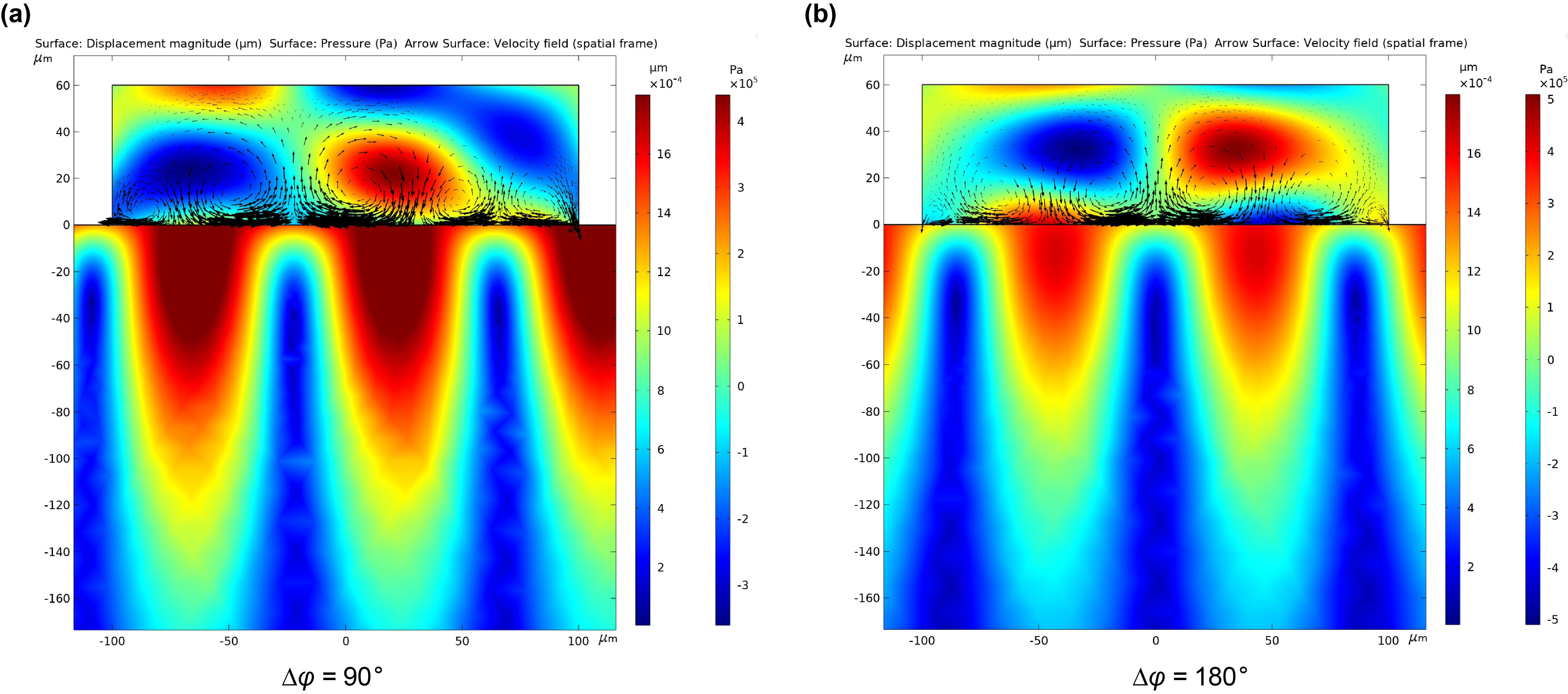


**Figure S4. Numerical simulation of applying phase difference between the two IDTs at (a) 90° (**$\Delta\varphi={90}^{^{\circ}}$**), and (b) 180° (**$\Delta\varphi={180}^{^{\circ}}$**).**


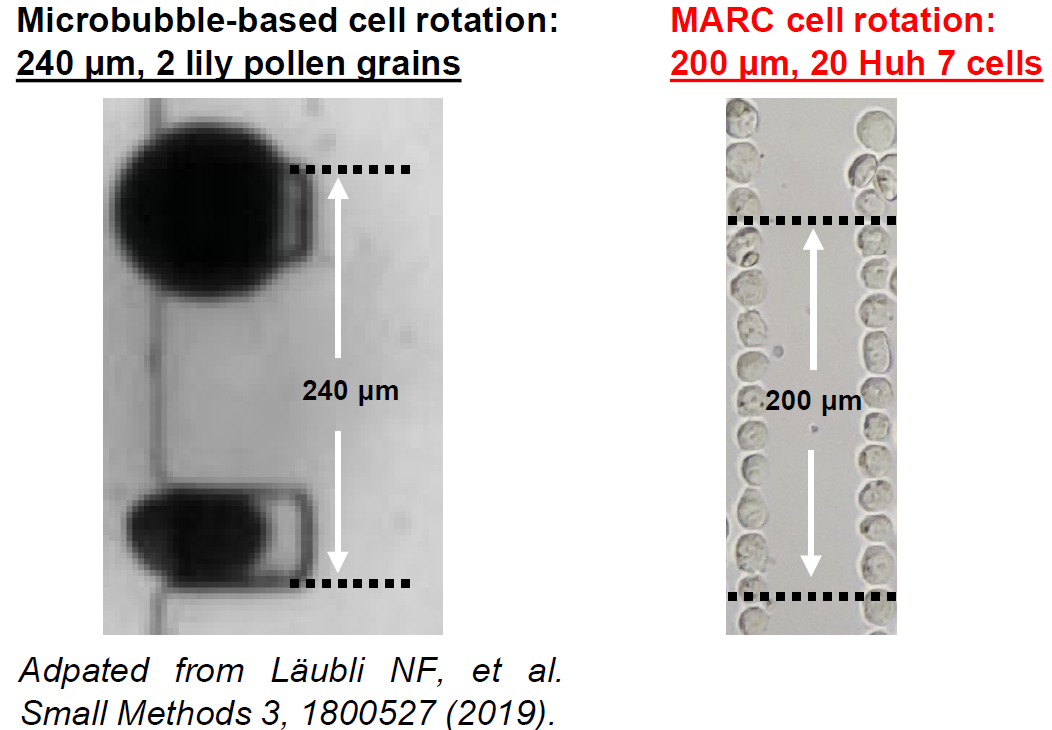


**Figure S5. Rotation throughput footprint comparison between a representative microbubble method and with MARC system.** Adapted with permission. [1] Copyright 2019, Wiley-VCH.


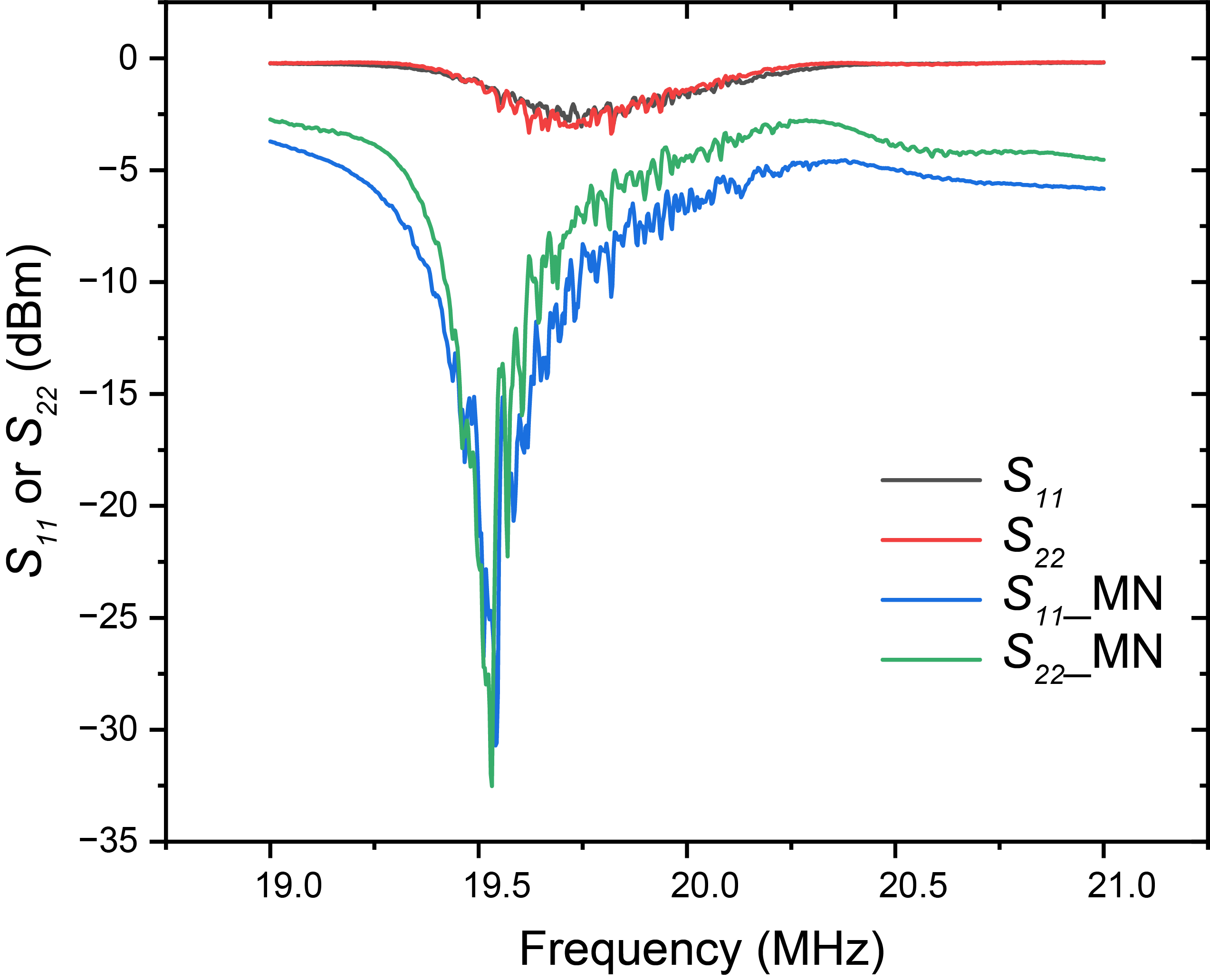


**Figure S6. Electrical characterisation of the *S*_11_ and *S*_22_ of the MARC with and without matching networks.**

**
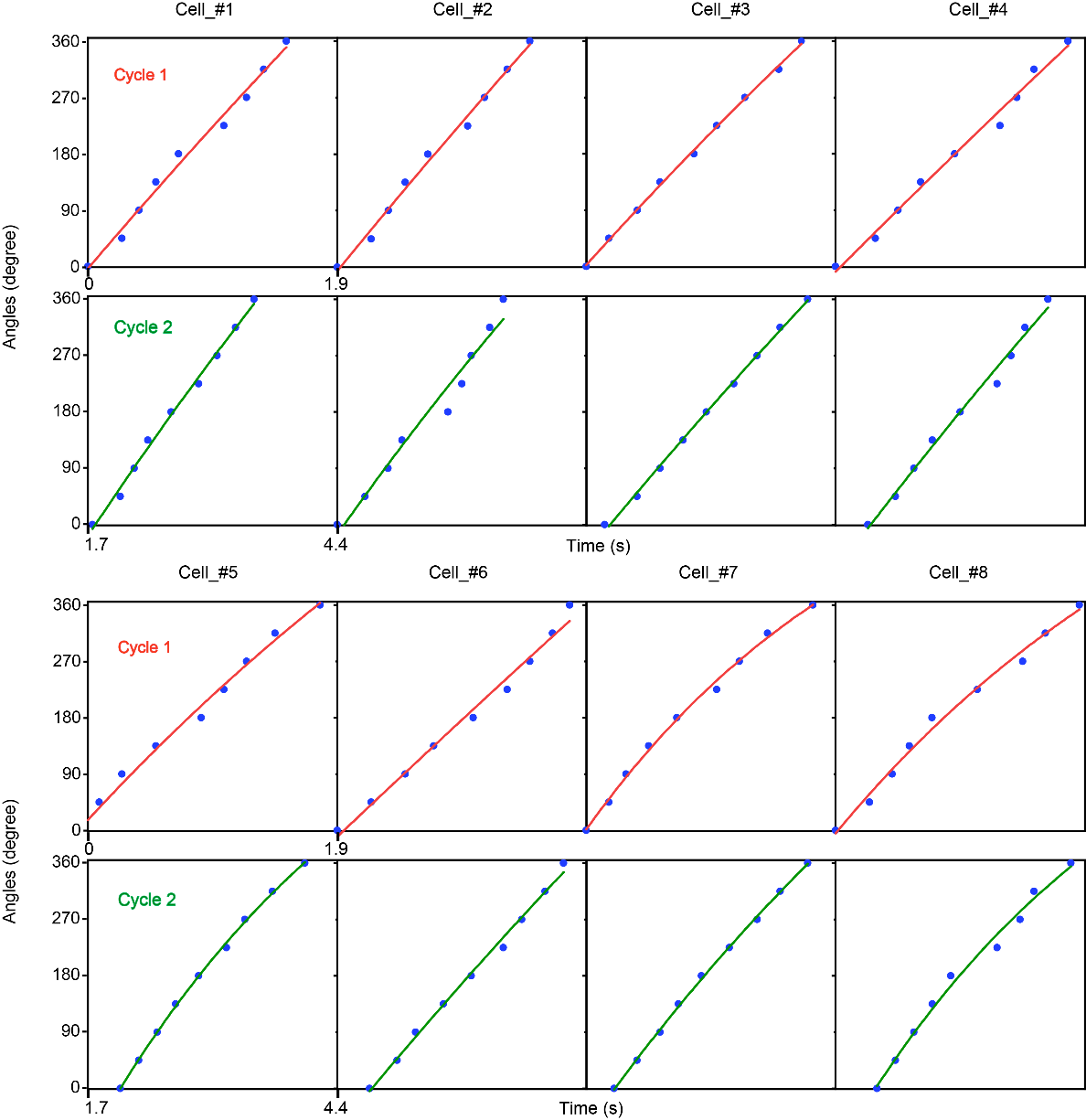
**

**Figure S7. Rotation angle quantification of the Huh7 cell (*n* = 8) during two rotation cycles (i.e., two times 360 degree) under 12.5 Vpp.**

**
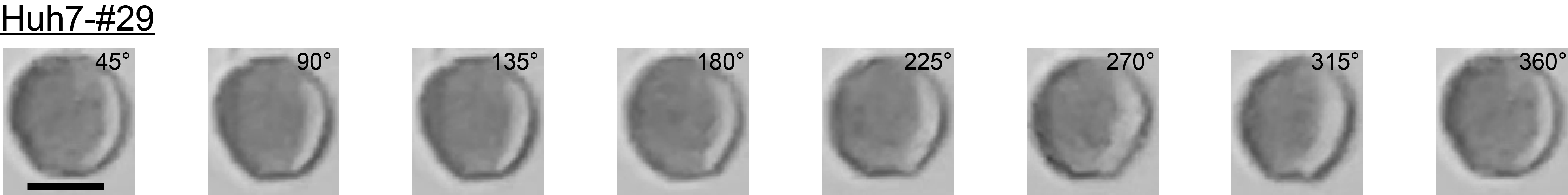
**

**Figure S8. The microscopic image of the Huh7-#29 during the rotation.** Scale bar, 10 μm.


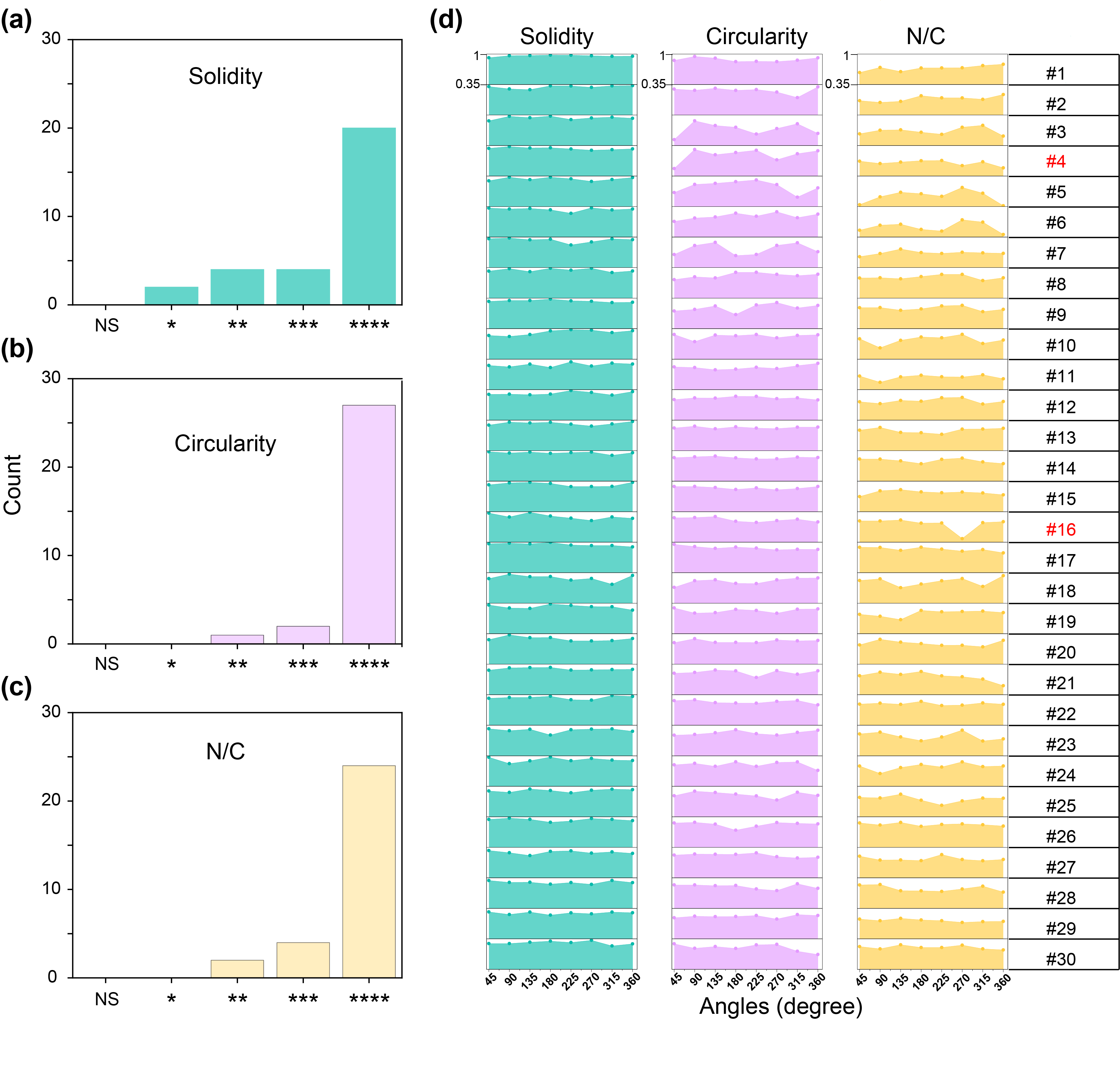


**Figure S9. Statistical analysis of the multi-angle impact on Huh7 cell cytopathology evaluation.** Significant difference analysis of nuclear **(a)** solidity, **(b)** circularity, and **(c)** N/C for single-cell during one circle rotation process. **(d)** The shaded connection lines of eight angles for each cell further visually reflect the significant effect of the multi-angle on the three nuclear parameters quantification (*n* = 30). All *p*-values were determined using one-way ANOVA (normally distributed) or Kruskal-Wallis tests (non-normally distributed). *NS*: no significance. *: *p* < 0.05, **: *p* < 0.01, ***: *p* < 0.001, ****: *p* < 0.0001.

**Table S1. Detailed geometry parameters of the PCB IDT and PDMS microchannel.**

| **PCB IDT** | IDT finger width | IDT finger space | wavelength | aperture | IDT pairs |
| --- | --- | --- | --- | --- | --- |
|  | 38.7±3.1 μm (Average±SD) | 61.1±3.0 μm (Average±SD) | 199.6±4.8 μm (Average±SD) | 10 mm | 40 |
| **PDMS microchannel** | width | height | length | PDMS thickness | |
|  | 200 μm | 60 μm | 15 mm | 5 mm | |

**Table S2. The measured rotational speed of Huh cells against the amplitude of the input signal from 7.5 Vpp to 17.5 Vpp.**

| **Input voltage (Vpp)** | **Cell rotation angular velocity (r.p.m., revolutions per minute)** | | | | | | | ***p* values of statistical analysis between trace 1 and trace 2.** (***NS*: no significance, *p* > 0.05)** |
| --- | --- | --- | --- | --- | --- | --- | --- | --- |
|  | **Cell trace 1** | | | **Cell trace 2** | | | |  |
|  | Mean | SD | *n* | | Mean | SD | *n* |  |
| 7.5 | 5.13333 | 0.73568 | 15 | | 5.23333 | 0.74141 | 15 | 0.77 (*NS*) |
| 10 | 14.8406 | 0.1327 | 15 | | 14.43242 | 0.06562 | 15 | 0.33 (*NS*) |
| 12.5 | 30.84364 | 0.06136 | 15 | | 30.16413 | 0.07201 | 15 | 0.54 (*NS*) |
| 15 | 46.21856 | 0.04907 | 15 | | 43.96559 | 0.09915 | 15 | 0.44 (*NS*) |
| 17.5 | 54.94048 | 0.05152 | 15 | | 56.01462 | 0.03356 | 15 | 0.65 (*NS*) |

**References**

[1] Läubli NF, *et al*. 3D Manipulation and Imaging of Plant Cells using Acoustically Activated Microbubbles. *Small Methods* 3, 1800527 (2019).

**Video S1. Two-column rotation of Huh7 cells.**

**Video S2.** **Stable high-throughput Huh7 cell rotation.**

**Video S3.** **Rotation of IHH-#1 and IHH-#2 cells to display the appearance nucleus outline even without any staining.**

**Video S4.** **Cell rotation speed comparison of Huh7 cells under 7.5 Vpp, 12.5 Vpp, and 17.5 Vpp.**
